# Enhanced Hydrolytic Stability of Siliceous Surfaces Modified with Pendant Dipodal Silanes

**DOI:** 10.1002/chem.201402757

**Published:** 2014-07-14

**Authors:** Barry Arkles, Youlin Pan, Gerald L Larson, Mani Singh

**Affiliations:** [a] Gelest Inc. 11 East Steel Rd. Morrisville, PA 19067 (USA) E-mail: executiveoffice@gelest.com

**Keywords:** dipodal silanes, hydrolytic stability, silicon, siloxane–silanol equilibria, surface chemistry

## Abstract

Dipodal silanes possess two silicon atoms that can covalently bond to a surface. They offer a distinct advantage over conventional silanes commonly used for surface modification in terms of maintaining the integrity of surface coatings, adhesive primers, and composites in aqueous environments. New nonfunctional and functional dipodal silanes with structures containing “pendant” rather than “bridged” organofunctionality are introduced. The stability of surfaces in aqueous environments prepared from dipodal silanes with hydrophobic alkyl functionality is compared to the stability of similar surfaces prepared from the conventional silanes. In strongly acidic and brine environments, surfaces modified with dipodal silanes demonstrate markedly improved resistance to hydrolysis compared to surfaces prepared from conventional silanes. Pendant dipodal silanes exhibit greater stability than bridged dipodal silanes. The apparent equilibrium constant for the formation of silanol species by the hydrolysis of a disiloxane bond was determined as *K*_c_=[SiOH]^2^/[Si-O-Si][H_2_O]=6±1×10^−5^ and is helpful in understanding the enhanced hydrolytic stability of surfaces modified with dipodal silanes.

## Introduction

Silanes that alter the chemical and physical properties of surfaces have been studied intensively for over sixty years.[1] Silanes utilized to modify surfaces are generally divided into two categories. “Functional” silanes or coupling agents are utilized for polymer composites,[2] biomolecule immobilization, and adhesive technologies. “Nonfunctional” silanes are employed to modify the surface energy or wettability of substrates and are not intended to impart chemical reactivity to the substrate. Together, both classes are often referred to as organofunctional silanes. Both categories rely on oxane bond formation with a hydroxyl functional substrate to effect the surface modification. The role of water is important in this chemistry since the vast majority of silane surface treatments are carried out by a hydrolytic deposition process and, after deposition, water adsorption at the interface is an important factor in determining the durability of the surface modification. The role of water in silane deposition and modification of surfaces is recognized in the generally accepted mechanism first proposed for silane deposition.[3]

The stability of properties associated with surface modification depends on maintaining tight equilibrium conditions in the physical region associated with the transition from the substrate to the matrix. The physical region that extends from the unmodified bulk phases of both the substrate and matrix is referred to as the interphase. The interphase region associated with silane modification is most properly viewed as a dynamic region in which a steep gradient in properties is mediated by the interaction of molecular water with oxane bonds. More specifically, the dynamics are driven by the hydrolysis and reformation of oxane bonds. Plueddeman[4] recognized this dynamic when considering that in composites for which there were substantial differences between the coefficient of thermal expansion between inorganic substrates and polymers physical properties were maintained during high-temperature processing and room-temperature performance. For example, mismatched coefficients of thermal expansion bond formation in a thermoplastic composite, such as nylon 6/6 and glass fiber, which processes at melt temperatures of 280–295 °C would have sufficient stress when cooled to room temperature to undergo interfacial failure if the bond between the glass fiber to the nylon was “fixed”. Separately, he observed that despite the fact that although the oxane bonds between most metals and silicon were known to be hydrolytically unstable, the silane surface modification properties of minerals was maintained. For example, despite the poor hydrolytic stability of titanium–oxygen–silicon bonds, literally thousands of tons of octylsilanes are effectively employed in modifying surface properties for white titanium oxide pigments in paints and coatings.

The success of silanes in numerous coupling applications is in large part due to the hydrolysis and reformation of oxane bonds between silanes and substrates, but at the same time hydrolysis without oxane bond reformation is a critical factor in their failure. It has been observed that overpolymerization of polymers onto substrates modified with silanes reduces the mobility of hydrolyzed silanes.[5] Long-term interfacial failure may be the eventual outcome of scission of the oxane bond coupled with diffusion of the silane from the interphase and a swelling or physical displacement of the resin from the original interphase region. Both the diffusion of silanols and displacement of the resin away from the inorganic substrate compromise system equilibrium and reduce the probability of reformation of oxane bonds.

This work explores two issues associated with the long-term failure of silane-modified inorganic substrates: the dynamism of siloxane bond hydrolysis and reformation and organofunctional silanes with dipodal structures, which reduce the probability of diffusion from the inorganic substrate.

**Scheme 1:**
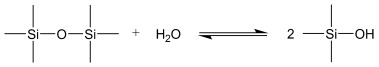
Siloxane–silanol equilibrium.

While the kinetics of siloxane bond formation in aqueous systems have been studied extensively, there exists only sparse data on the equilibrium constant for siloxane bond formation and hydrolysis in aqueous media (Scheme 1).

This is presumably primarily because the condensation of silanol species results in species with siloxane bonds that are generally insoluble in water and form bulk insoluble phases that do not readily participate in system equilibria. Silicic acid and trimethylsilanol are examples of species that form bulk phases, silica and hexamethyldisiloxane, respectively, that do not readily participate in equilibrium studies in neutral aqueous media.[6–7] Further, the low concentrations of silicic acid normally found in water, typically 50–100 ppm, leads to the impression that the equilibrium favors the formation of the siloxane bond. However, even the determination of equilibrium silicic acid concentration in water is subject to considerable discussion.[8]

On the other hand, phenomena, such as Ostwald ripening of Stober process silica particles and formation of monolithic structures by sol–gel processing, suggests that the equilibrium is much more balanced.[9–10] Studies of the stability of water soluble organosiloxanes have been limited to surfactants with poly(ethyleneoxy) linkages and, while these materials degrade within hours in aqueous systems,[11] the degradation is typically associated with the oxidatively induced degradation of poly(ethylene oxide) segments rather than hydrolysis of the siloxane segments.

Several factors can potentially interfere with a straightforward determination of a silanol–siloxane equilibrium constant. Ionization of silanol groups can take place at near neutral pH, the same conditions of interest for studying the silanol–siloxane equilibrium. Other considerations that may preclude simple determination of the silanol–siloxane equilibrium constant is that most compounds of interest, such as silicic acid and dimethylsilanediol, can form multiple siloxane bonds and undergo polymerization that would influence the p*K*a of remaining silanol groups. Nevertheless, an understanding of the apparent equilibrium between siloxane and silanol species would be helpful in understanding long-term interphase behavior.

The objective of this study was to improve the durability of organofunctional silane surface modifications by increasing the number of potentially available sites for an organosilane to form oxane bonds either to a substrate or to form silsesquioxane polymers. It was of further interest to determine whether the proximity of the bonding sites within an organofunctional silane had an effect on the durability of silane surface modification under aqueous conditions. To provide a proper context for the potential of enhancing the hydrolytic stability of organofunctional silane surface treatments, the apparent equilibrium constant for a stable water soluble disiloxane was determined.

## Results and Discussion

**Scheme 2:**
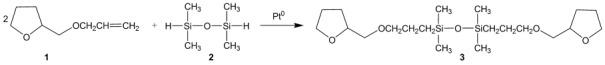
Hydrosilylation of 2-(allyloxymethyl)tetrahydrofurane with tetramethyldisiloxane.

**Scheme 3:**
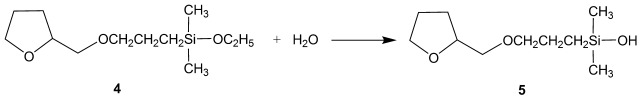
Hydrolysis of tetrahydrofurfuryloxypropyldimethylethoxysilane.

As probes for the stability of the Si–O–Si bonds, an organofunctional disiloxane and an organofunctional silanol were synthesized by hydrosilylation of tetramethyldisiloxane and dimethylethoxysilane according to methods previously reported.[12] Both compounds readily dissolved in water and no tendency toward phase separation was observed. In the case of the tetrahydrofurfuryloxypropyldimethylethoxysilane, ethanol formed by hydrolysis was removed by azeotropic distillation prior to the equilibrium studies (Schemes 2 and 3)

**Scheme 4:**
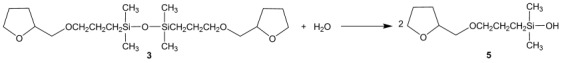
Hydrolysis of 1,3-bis(tetrahydrofurfuryloxypropyl)tetramethyldisiloxane.

In independent experiments, both the disiloxane and the silanol were evaluated in neutral aqueous systems and, after equilibration, the relative amounts of silanol and disiloxane were determined (Scheme 4).

Various organofunctional silanes were prepared containing two silicon atoms with alkoxy substituents. Compounds with substitution in the alpha and omega position have sometimes been referred to as “bis(silanes)” and have been the subject of much investigation, particularly in the field of metal coatings and primers.[13–15] We have chosen to call this class of silanes “dipodal silanes” from the Greek in which “two feet” can be fixed to a substrate. Dipodal silanes can be further differentiated in a general sense as to whether they are “bridged” dipodal silanes or as “pendant” dipodal silanes. For the most part, dipodal silanes utilized both commercially and reported in the literature may be regarded as “bridged”, that is the silane substitution is at terminal ends of an organic moiety. Examples of non-functional bridged dipodal silanes are 1,10-bis(trimethoxysilyl)decane and 1,8-bis(triethoxysilyl)octane. Examples of functional bridged dipodal silanes are bis(trimethoxysilylpropyl)amine and bis(triethoxysilylpropyl)tetrasulfide. Pendant dipodal silanes, which possess substrate reactive sites separated by only one or two carbon atoms, afford an organic functionality that extends from the substrate in a manner similar to traditional or “conventional” organofunctional silanes. Representative examples of classes of organofunctional silanes are depicted in structures **6**–**9**.
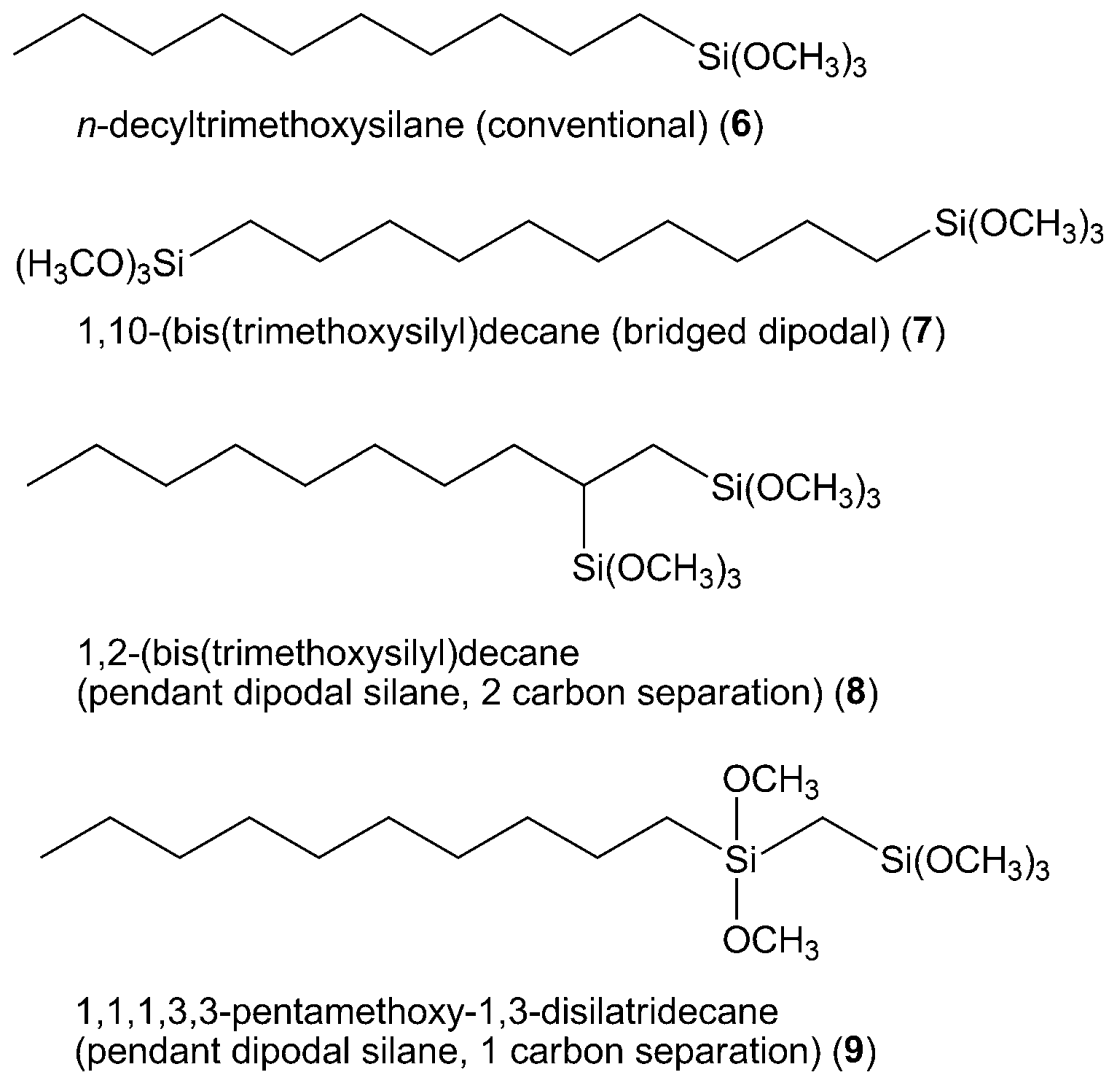


**Scheme 5:**
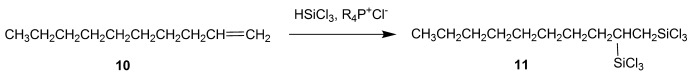
Disilylation of decene with trichlorosilane.

Two synthetic methods were utilized to generate the pendant dipodal silanes evaluated in this study. The pendant silanes with two-carbon separation were synthesized by a disilylation method as reported earlier (Scheme 5).[16]

**Scheme 6:**
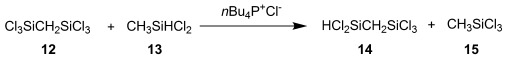
Redistribution of bis(trichloro)methane with methyldichlorosilane.

**Scheme 7:**
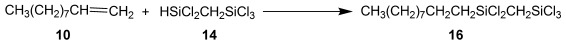
Hydrosilylation of decene with 1,1,1,3,3-pentachloro-1-3-disilapropane.

**Scheme 8:**
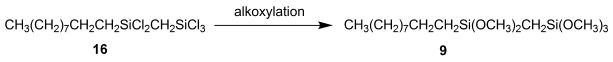
Alkoxylation of 1,1,1,3,3-pentachloro-1-3-disilatridecane.

The syntheses of the pendant silanes with a one-carbon separation were achieved in three steps, by starting with a redistribution reaction, followed by hydrosilylation and then esterification or esterification followed by hydrosilylation (Schemes 6, 7, and 8).[17].

Examples of both functional and nonfunctional silanes were prepared. Evaluation of nonfunctional silane surface treatments in aqueous systems was considered to be a better probe than functional silanes for hydrolytic stability. A functional silane anchored by reaction with an organic component in a composite coating would be restrained from diffusing from the interphase and, thus, exhibit extended time to failure. Further, contact-angle studies provide a simple metric that is readily related to the presence of a silane, whereas mechanical properties associated with composites and coatings are less direct.

### Apparent equilibrium constant of the siloxane bond in water

Initial and equilibrium concentrations of 1,3-bis(tetrahydrofurfuryloxypropyl)tetramethyldisiloxane and tetrahydrofurfuryloxypropyldimethylsilanol in solutions of water and THF were determined by GC analysis (with adjustments for response factors) and summarized in the Tables 1 and 2. These reagents were selected because they were soluble in water at all concentrations. 1,3-Bis(tetrahydrofurfuryloxypropyl)tetramethyldisiloxane was used as the starting material in Table 1. (Tetrahydrofurfuryloxypropyl)dimethylsilanol was used as a starting material in Table 2. The mixtures were stirred in borosilicate glass reactors at room temperature, and reached equilibrium in no longer than 30 days. Unlike polyethyleneoxy-substituted siloxanes, the formation of low molecular weight species associated with oxidative scission of C–O–C bonds was not observed.

**Table 1:** Initial and final of concentration of siloxane and silanol derivatized from disiloxane

Components	Disiloxane [mol]		Water [mol]		Silanol [mol]	
	Initial	Final	Initial	Final	Initial	Final
1	0.1000	0.0999	0.5000	0.4999	0.0000	0.0018
2	0.5000	0.4983	0.5000	0.4983	0.0000	0.0033
3	0.1000	0.9927	1.0000	0.9927	0.0000	0.0014

**Table 2:** Initial and final of concentration of siloxane and silanol derivatized from silanol

Components	Disiloxane [mol]		Water [mol]		Silanol [mol]	
	Initial	Final	Initial	Final	Initial	Final
1	0.0000	0.0773	0.5000	0.4991	0.1564	0.0163
2	0.0000	0.1293	0.6518	0.6509	0.2607	0.0019
3	0.0000	0.1294	0.5000	0.4991	0.2607	0.0016

The apparent equilibrium constant for the hydrolysis of one mole of disiloxane to two moles of silanol was calculated for each data set and then averaged, providing the result:


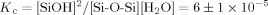
(1)

The apparent equilibrium constant of 6×10^−5^ is consistent with the dynamism suggested for the siloxane bond in surface modification applications. While long-term hydrolytic stability studies of polymeric siloxane and silica bulk phases are not relevant to the equilibrium solution studies of this investigation, they provided a perspective that suggested that the siloxane bond is less susceptible to hydrolysis than reality. The preservation of the siloxane (Si–O–Si) bond in aqueous systems is significantly more favored than the silicon ester (Si–O–C) bond that has been estimated at 2×10^−2^.[18]

The apparent equilibrium constant of the siloxane bond is significant when considering the design of new functional silane structures. The simplest case for most conventional silane surface modifications is that only one of the three intermediate hydroxyl groups on each silicon atom formed during hydrolytic deposition condenses with a silanol on a siliceous substrate to form a siloxane bond. The remaining silanols condense into the silsequioxane structures of the polymeric interphase or remain as unbound or hydrogen bonded silanols. Considering this simplest case, it can be argued that, in contrast to the organofunctional silanol species that are subject to diffusion, the silanol of the substrate is fixed and unable to leave the equilibrium system. Thus, a factor of ≈10^−2^ is a better metric than ≈10^−4^ (rounding 6×10^−5^) of the apparent equilibrium constant. Disregarding the formation of polymeric silsesquioxanes, a projected improvement in stability for a surface modification on a silane with two silicon atoms and thus two opportunities to form siloxane bonds could potentially improve the durability of the bond 100-fold. If all of the potential siloxane bonds of each silicon atom are considered, then the probability of hydrolysis followed by diffusion from the substrate would be decreased 10^−2^×10^−2^×10^−2^, resulting in a 1×10^6^-fold more stable structure. This discussion is overly simplified, but appears to be consistent with improvements in the hydrolytic stability observed with dipodal silanes. Significant factors that clearly would have an impact on the hydrolytic stability of a specific system are: 1) the solubility of the monomeric silanol species, the tendency of these materials to form POSS structures, and the distance between silicon atoms in a dipodal silane and 2) water transport to the interphase and adsorption on the substrate.

### Synthesis of dipodal silanes

**Scheme 9:**
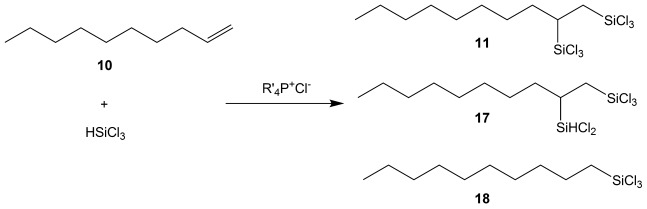
Disilylation of decene with trichlorosilane.

Both conventional and bridged dipodal silanes were prepared in high yield by well-established protocols for reacting trichlorosilane with terminal olefins in the presence of a Pt^0^ catalyst. Pendant dipodal silanes with a two-carbon bridge were prepared from terminal olefins and trichlorosilane in the presence of a tetraalkylphosphonium chloride catalyst that exhibited liquid behavior at room temperature (Scheme 9).

**Figure 1:**
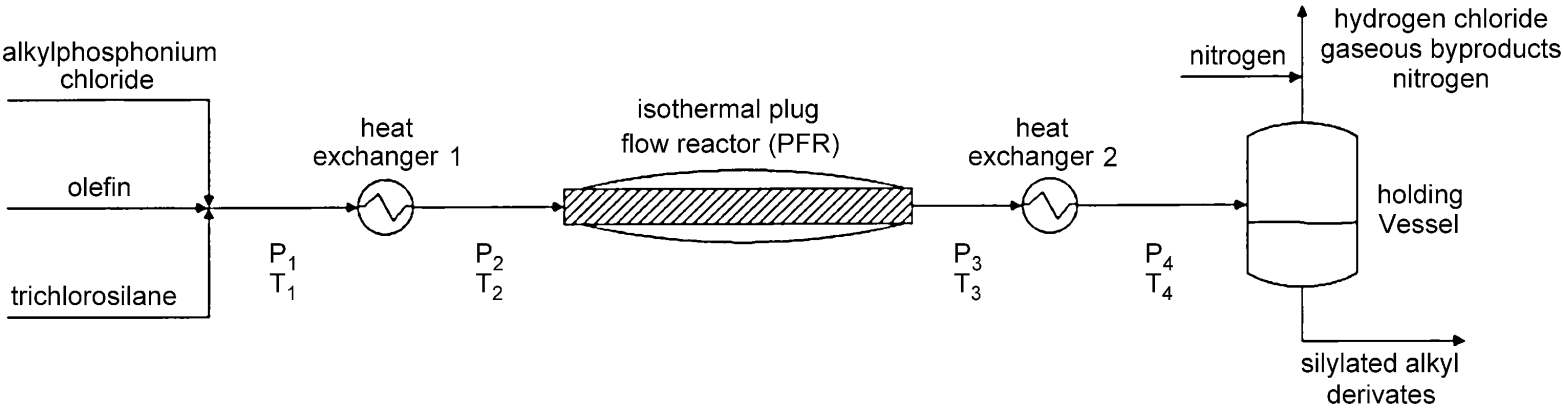
Schematic of apparatus for continuous high-pressure disilylation. Exemplary conditions for 1,2-bis(trichlorosilyl)decane: Catalyst (*n*-tetradecyl)tri-*n*-butylphoshonium chloride; mole ratio decene/trichlorosilane/R_4_P^+^Cl^−^ 1:2:0.1; reaction temperature: 180°C; residence time: 2 h; pressure: 23 atm.; yield: 84 %; byproducts: 1-(trichlorosilyl)-2-(dichlorosilyl)decane, trichlorosilyldecane, SiCl_4_, H_2_SiCl_2_.

The reaction conditions were optimized by independently varying co-feed ratios of reactants and catalyst into a continuous high pressure reactor, which allowed for greater temperature control and continuous venting of gaseous byproducts as depicted in Figure 1.

The data presented in Table 3 indicates that this procedure proceeds in significantly higher yield than previously reported for the analogous static reaction utilizing tetrabutylphosphonium chloride as a catalyst,[19] producing >80 % of the desired product in preference to the partially reduced disilylated byproduct and single hydrosilylation byproduct. The structures, yields, and physical properties of dipodal silanes prepared are summarized in Tables 3–5.

**Table 3:** Nonfunctional pendant silanes (with 2 carbon separation)

Organic	Isolated yield [%]	B.p. [°C/mmHg]	Density [g/cc, 20 °C]
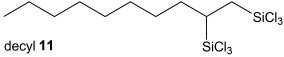	87	120–2°/1	1.250
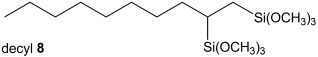	95	130–2°/0.4	0.984
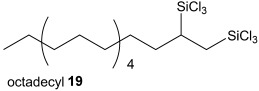	71	186–9°/0.2	1.103

**Table 4:** Nonfunctional pendant silanes (with one carbon separation)

Organic	Isolated yield [%]	B.p. [°C/mmHg]	Density [g/cc, 20 °C]
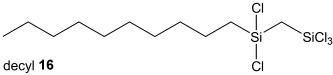	59	126/0.1	1.178
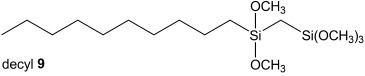	46	123/0.1	0.955
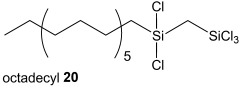	91	192–4/0.2	1.059
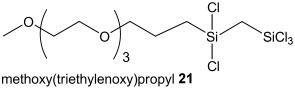	70	170–2/0.4	1.262
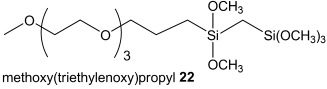	56	170–2/0.1	0.994

**Table 5:** Functional pendant silanes (with one carbon separation)

Organic	Isolated yield [%]	B.p. [°C/mmHg]	Density [g/cc, 20 °C]
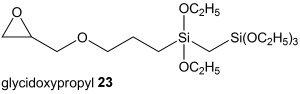	15	168–78/0.5	1.003
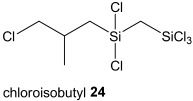	60	89–91/0.4	1.380
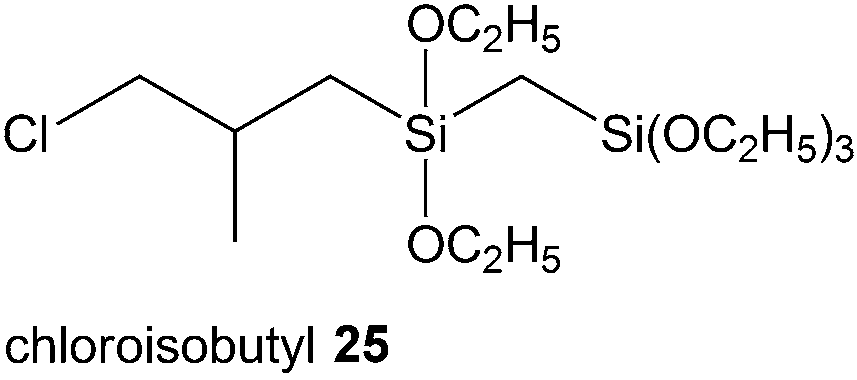	60	110/0.5	1.020
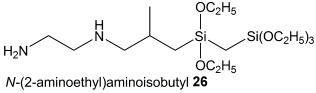	89	130–50/0.5	0.990

### Behavior of surfaces treated with dipodal silanes

Silane treatment contact angle measurements (sessile, advancing, and receding) for borosilicate glass slides treated with representative silanes (**6**–**9**) are listed in Table 6. All the silanes, except 1,10-bis(trimethoxysilyl)decane, show sessile water contact angles (CA) ≥90°. 1,10-Bis(trimethoxysilyl)decane has two trialkoxysilyl groups bridged by a long alkyl chain and, therefore, does not possess terminal methyl groups that contribute towards low surface energy (hydrophobic) behavior. All the silane coatings show a hysteresis (advancing CA–receding CA)>10°, which suggests the possibility of surface defects or inhomogeneities, but examination by SEM revealed smooth homogeneous coatings.

**Table 6:** Initial water contact angles for the various silanes

Silane	Water contact angle [°]		
	Sessile	Advancing	Receding
**6**	94±1	104±1	91±2
**8**	102±1	106±2	95±1
**9**	104±2	110±1	98±2
**7**	78±2	84±1	73±2

**Figure 2:**
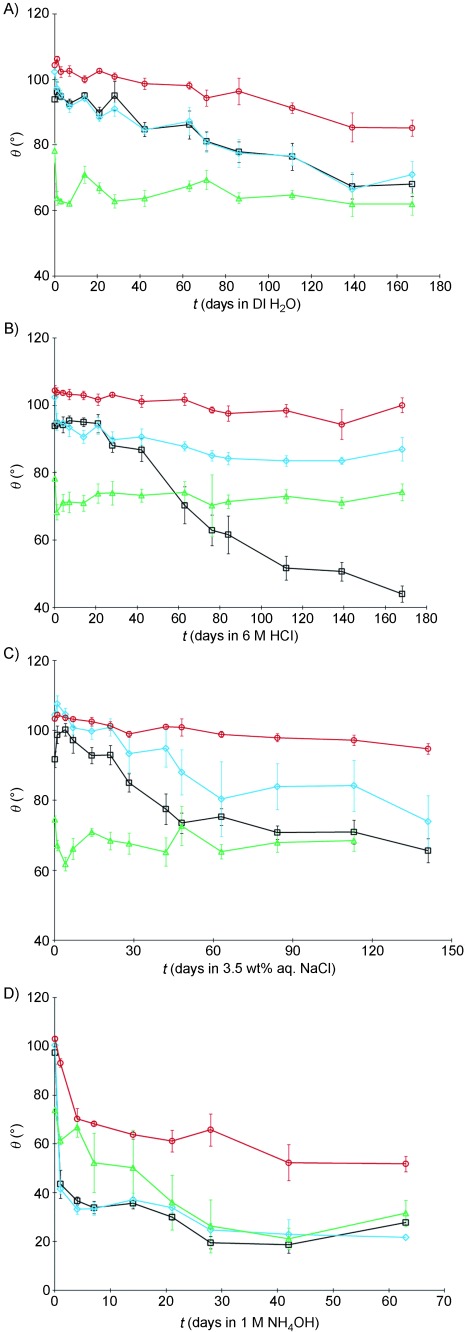
Sessile water contact-angle measurements for deionized water (A), 6 m HCl (B), 3.5 % aqueous NaCl (C), and 1 m NH_4_OH (D) (□: n-decyltriethoxysilane (6); ◊: 1,2-bis(trimethoxysilyl)decane (7); ○: 1,1,1,3,3-pentamethoxy-1,3-disilatridecane (8); ▵: 1,10-bis(trimethoxysilyl)decane (7)).

Static immersion durability tests for silanes (**6**–**9**) were conducted in deionized water, 6 m HCl, 3.5 % aqueous sodium chloride and 1 m NH_4_OH at room temperature. The integrity of the coating is directly related to changes in the water contact angle and an increase in contact angle hysteresis measured on the substrates. Figures 2A–D show the progression of the sessile water contact angle with time for the substrates coated. In cases for which the contact angle hysteresis exceeded 30°, the data was judged to not be meaningful and testing was ended.

Deionized water immersion is relatively mild in comparison to the other aqueous media evaluated. The ≡Si-O-Si≡ linkage is very susceptible to hydrolysis at pH<3 and >8, with basic conditions significantly more deleterious than acidic conditions. The static immersion test in 6 m HCl for silane-modified surfaces is an extreme condition and acts as an accelerated acidic durability test. 1,2-Bis(trimethoxysilyl)decane, and 1,1,1,3,3-pentamethoxy-1,3-disilatridecane form the most durable coatings as the static water contact angle after 60 days in HCl is 102±2 and 94±3°, respectively. The conventional silane, *n*-decyltriethoxysilane, and the pendant dipodal, 1,2-bis(trimethoxysilyl)decane contact angles follow each other very closely up to day 28, after which the conventional silane coating starts to significantly degrade. The contact angles drop from 102±1 on day zero to 88±1° on day 60 for *n*-decyltriethoxysilane. Similar behavior is seen in the static immersion studies in deionized water for all the silane treatments. The rate of deterioration of the silane coatings in 3.5 % sodium chloride was greater than anticipated. The bridged dipodal silane, 1,10-bis(trimethoxysilyl)decane, which appeared to maintain a relatively stable contact angle over time in the first part of the experiment, began to exhibit greater hysteresis and a mottled appearance, with hysteresis exceeding 30° after 120 days. Of all the silanes, 1,1,1,3,3-pentamethoxy-1,3-disilatridecane formed the most durable coating in all environments tested and, notably, was the only silane to form coatings with even nominal resistance to 1 m NH_4_OH. Resistance to hydrolysis of organofunctional silanes appears to be optimized when there is a relatively small number of carbon atoms between the silicon centers.

## Conclusion

Dipodal silanes possess two silicon atoms that can covalently bond to a surface. New nonfunctional and functional dipodal silanes with structures containing “pendant,” rather than “bridged,” organofunctionality were synthesized. Both pendant and bridged silanes offer a distinctive advantage over conventional silanes in terms of maintaining the integrity of surface coatings, adhesive primers and composites in aqueous and aggressive environments. The improved durability of such dipodal silanes is associated with an increased cross-link density of the interphase and the inherent resistance to hydrolysis, as they can potentially form five or six, rather than three, oxane bonds. Pendant dipodal silane structures are more resistant to hydrolysis than the bridged dipodal silane structure with single-carbon separated (disilapropyl)silanes demonstrating the greatest resistance to hydrolysis and best stability.

## Experimental Section

### General considerations

All synthetic reactions were run under an atmosphere of purified nitrogen. Trichlorosilane, dichloromethylsilane and the various olefins were of a minimum 98 % purity, and were used without further purification. ^1^H NMR spectra were recorded on a JEOL 400 MHz instrument. The INEPT sequence was utilized in collecting ^29^Si NMR spectroscopic data. *n*-Decyltrimethoxysilane was a Gelest commercial product.

### Preparation of 1,1,1,3,3-pentachloro-1,3-disilapropane (14)

A 5-gallon autoclave equipped with a stirrer, pot thermometer and addition ports was charged with 1,1,1,3,3,3-hexachloro-1,3-disilapropane (9901.5 g; 35 mol), dichloromethylsilane (4428.7 g; 38.50 mol), and tri(*n*-hexyl)(*n*-tetradecyl)phosphonium chloride (258.0 g, 0.88 mol) The reaction mixture was heated to between 80 and 90° for 10 h. The reaction temperature was cooled to room temperature and the crude reaction product discharged into a 12-liter flask. Vacuum was applied to remove volatiles and the residue was distilled at 6 mm Hg and a pot temperature of 60 °C. This served to remove various impurities and produce a mixture of 1,1,1,3,3-pentachloro-1,3-disilapropane and 1,1,1,3,3,3-hexachloro-1,3-disilapropane in a ratio of 46:54. This material was used for the hydrosilylation experiments to prepare the 1,3-disilaalkane derivatives.

### Preparation of 1,1,1,3,3-pentaethoxy-1,3-disilapropane

A 5-liter flask equipped with condenser, dropping funnel, and magnetic stirrer was charged with of 1,1,1,3,3-pentachloro-1,3-disilapropane (600 g; 1.11 mol active) prepared above. Triethylorthoformate (8200 g; 55.5 mol) was added dropwise with the reaction temperature at 90°. A gas (ethyl chloride) was evolved. After the evolution of gas had ceased the temperature was raised to 120 °C and triethylorthoacetate (820 g; 5.55 mol) was added dropwise to finish the esterification. Chloride analysis indicated that the reaction was complete. After completion of the reaction, vacuum was applied to remove the more volatile components. Distillation gave 270 g (42.9 %) of the title product as a 44:56 mixture of 1,1,1,3,3-pentaethoxy-1,3-disilapropane and 1,1,1,3,3,3-hexaethoxy-1,3-disilapropane. B.p. 88–103 °C/1.5 mmHg. This mixture was used for the hydrosilylation experiments.

### Preparation of 1,2-bis(trichlorosilyl)decane (11)

A 5-gallon autoclave equipped with a stirrer, pot thermometer, and addition ports was charged with a premix of 1-decene (1616.5 g; 11.5 mol), trichlorosilane (4683.2 g; 34.6 mol), and a 50 % toluene solution of (tetradecyl)tri-*n*-butylphosphonium chloride (679.7 g; 2.3 mol). The reaction mixture was slowly heated to 220 °C and held at that temperature for 18 h. The reaction temperature was cooled to room temperature and the crude reaction product discharged into a 12-liter flask. Distillation of the product gave 3684.7 g (87 %) of the title dipodal silane. B.p. 120–2 °C/0.2 mmHg; density, 20 °C: 1.249 g/cc; ^1^H NMR (400 MHz): *δ*=1.88–1.70 (m, 3 H), 1.66–1.56 (m, 1 H), 1.56–1.36 (m, 2 H), 1.36–1.18 (m, 11 H), 0.88 ppm (t, 3 H).

### Preparation of 1,2-bis(trimethoxysilyl)decane (8)—representative esterification procedure

A 22-liter flask equipped with condenser, dropping funnel, and overhead stirrer was charged with (6,463 g, 17.25 mol) of 1,2-bis(trichlorosilyl)decane and heated to about 100 °C. Trimethylorthoformate (7,322 g; 69.0 mol) was charged to the addition funnel and added dropwise at such a rate so as to control the evolution of the chloromethane and methyl formate. The progress of the reaction was followed by measurement of the pH with a pH of 6–7 indicating full reaction of the chlorosilane. To finish the reaction, trimethylorthoacetate (5,181 g; 43.13 mol) was added dropwise and the reaction temperature increased to 120 °C. After the reaction was complete the volatiles and excess *ortho* esters were removed at atmospheric pressure up to a pot temperature of 160 °C. Reduced-pressure distillation gave 5610.0 g (85 %) of the title product. B.p. 130–132°/0.4 mmHg; density, 20 °C: 0.984 g/cc; ^1^H NMR (400 MHz): *δ*=3.54 (s, 9 H), 3.51 (s, 9 H), 1.50–1.30 (m, 4 H), 1.25 (br s, 10 H), 1.05–0.98 (m, 1 H), 0.90–0.81 (m, 4 H), 0.46 ppm (dd, 1 H); ^29^Si{^1^H} NMR ( 400 MHz, CDCl_3_): *δ* =−44.41, −41.95 ppm.

### Preparation of 1,2-bis(trichlorosilyl)octadecane (19)

A procedure similar to that for the preparation of 1,2-bis(trimethoxysilyl)decane employing 1-octadecene (2,200 g; 9.0 mol) and trichlorosilane (3,600 g; 27 mol) gave 3257.6 g (71 %) of 1,2-bis(trichlorosilyl)octadecane as colorless liquid. B.p. 190 °C/0.1 mmHg; density, 20 °C: 0.923 g/cc; ^1^H NMR (400 MHz): *δ*=1.88–1.72 (m, 4 H), 1.60 (dd, 1 H), 1.54–1.36 (m, 2 H), 1.36–1.20 (m, 25 H), 0.88 ppm (t, 3 H).

### 1,1,1,3,3-Pentachloro-1,3-disilatridecane (16)

Under a nitrogen atmosphere, a 1-liter, 3-necked flask equipped with a heating mantle, magnetic stirrer, pot thermometer, addition funnel, and condenser was charged with 276.8 g of a mixture of approximately 46 % (dichlorosilylmethyl)trichlorosilane and 54 % bis(trichlorosilyl)methane [molar ratio 1:1]. The mixture was heated to 90 °C and 10 g of 1-decene were added to the mixture by an addition funnel, followed by 0.5 mL of 5 % hexachloroplatinic acid in tetrahydrofuran. An immediate exotherm was observed and the reaction mixture changed from clear to dark brown. An additional 173 g of 1-decene was added at an appropriate rate to maintain the temperature between 90 and 110 °C. Upon completion of the addition, an additional 0.25 mL of 5 % hexachloroplatinic acid solution was added and the mixture was stirred at 100 °C for 45 min. GC analysis of the mixture indicated the presence of some unreacted pentachlorodisilapropane. An additional 10 g of 1-decene were added and the reaction mixture was stirred an additional 45 min at 100°. Distillation provided 242 g (59 %) of the title compound. B.p. 126°/0.1 mmHg; density, 20 °C: 1.178 g/cc; ^1^H NMR (400 MHz): *δ*=1.58–1.48 (m, 4 H), 1.46–1.34 (m, 2 H), 1.34–1.20 (m, 14 H), 0.88 ppm (t, 3 H).

**Preparation of 1,1,1,3,3-pentamethoxy-1,3-disilatridecane (9)**

The product from above was reacted according to the representative esterification procedure to give an 82 % yield of **9**. B.p. 123–4 °C/0.1 mmHg, density, 20 °C: 0.955 g/cc; ^1^H NMR (400 MHz): *δ*=3.55 (s, 9 H), 3.50 (s, 6 H), 1.40–1.15 (m, 16 H), 0.85 (t, 3 H), 0.70–0.60 (m, 2 H), −0.05 ppm (s, 2 H); ^29^Si{^1^H}( 400 MHz, CDCl_3_): *δ* −41.85, −4.22 ppm.

### Preparation of 1,1,1,3,3-pentachloro-1,3-disilaheneicosane (20)

A 2-liter flask equipped with heating mantel, magnetic stirrer, pot thermometer, addition funnel, and a dry-ice condenser was charged with the 1,1,1,3,3-pentachloro-1,3-disilapropane/1,1,1,3,3,3-hexachloro-1,-disilapropane mixture (276.8 g; 0.51 mol active reagent). The reaction mixture was warmed to 90 °C and 5 % chloroplatinic acid in THF (0.5 g) was added followed by the addition of 10 g of 1-octadecene. An immediate exotherm was observed indicating initiation of the hydrosilylation. The remainder of the 187 g (0.72 mol) of 1-octadecene was added at a rate to maintain the reaction temperature between 90 and 110 °C. After the addition was complete, an additional 0.25 mL of the platinum catalyst solution was added and the reaction mixture was heated at 100 °C for an additional 45 min. Distillation provided 229 g (91 %) of the title product. B.p. 192–4 °C/0.2 mmHg; density, 20 °C: 1.059 g/cc; ^1^H NMR (400 MHz): *δ*=1.57–1.49 (m, 4 H), 1.42–1.35 (m, 2 H), 1.34–1.20 (m, 30 H), 0.88 ppm (t, 3 H).

### Preparation of [2-methoxy(triethyleneoxy)propyl]-1,1,1,3,3-pentachloro-1,3-disilapropane (21)

Under an atmosphere of nitrogen, a standard-equipped 500 mL flask was charged with allyloxytriethyleneoxymethyl ether (51.1 g; 0.25 mol). A 46:54 mixture of 1,1,1,3,3-pentachloro-1,3-disilapropane and 1,1,1,3,3,3-hexachloro-1,3-disilapropane (141.8 g; 0.26 mol of reactive silane) was placed in an addition funnel and ca. 25 g of the mixture containing **14** was added to the reaction mixture. The reaction mixture was heated to 85 °C and a 5 % THF solution of chloroplatinic acid (0.25 mL) was added. An immediate exotherm was observed with the solution changing from clear and colorless to dark brown. The balance of the chlorosilane mixture was added at a rate so as to maintain a reaction temperature of 85 to 105 °C. Upon completion of the silane addition, an additional 0.25 mL of the catalyst solution was added and the reaction mixture heated at 95° for 45 min. Distillation provided 80 g (70 %) of the title organosilane. B.p. 170–2 °C/0.5 mmHg; density, 20 °C: 1.262 g/cc; ^1^H NMR (400 MHz): *δ*=3.72- 3.56 (m, 8 H), 3.65–3.48 (m, 4 H), 3.17 (t, 2 H), 3.41 (s, 3 H), 1.71–1.59 (m, 4 H), 0.73–0.68 ppm (m, 2 H).

### Preparation of [2-methoxy(triethyleneoxy)propyl]-1,1,1,3,3-pentaethoxy-1,3-disilapropane (22)

In a one-pot, two-step process based on the above, allyloxytriethyleneoxymethyl ether (204 g; 1 mol) was hydrosilylated and the crude pentachloro derivative converted directly to the ethyl ester to give after distillation 310 g (62 %) of the title organosilane. B.p. 176–8 °C/0.1 mmHg: density, 20 °C: 0.994 g/cc; n_D_^20^=1.4359; ^1^H NMR (400 MHz): *δ*=3.80–3.65 (m, 10 H), 3.62- 3.56 (m, 8 H), 3.55–3.48 (m, 4 H), 3.37 (t, 2 H), 3.31 (s, 3 H), 1.68–1.59 (m, 2 H), 1.19–1.10 (m, 15 H), 0.65–0.58 (m, 2 H), −0.09 ppm (s, 2 H).

### Preparation of 1,1,1,3,3-pentaethoxy-1,3-disilahexylglycidyl ether (23)

A 1-liter flask equipped with condenser, addition funnel, magnetic stirrer, and pot thermometer was charged with the 44:56 mixture of 1,1,1,3,3-pentaethoxy-1,3-disilapropane and 1,1,1,3,3,3-hexaethoxy-1,3-disilapropane (283.75 g; 0.42 mol of active) prepared above and phenothiazine (0.04 g). The reaction mixture was heated to 90 °C and allylglycidyl ether (43.3 g; 0.38 mol) was added keeping the temperature between 85 and 115 °C. The reaction mixture was subjected to distillation through a short Vigreaux column with three successive distillations giving 100 g (64.5 %) of the title product in 96 % purity. B.p. 125–30 °C/0.1 mmHg; ^1^H NMR (400 MHz): *δ*=3.82–3.71 (m, 10 H), 3.65 (dd, 1 H), 3.48–3.31 (m, 3 H), 3.12–3.08 (m, 1 H), 2.75 (t, 1 H), 3.59–3.54 (m, 1 H), 1.69–1.61 (m, 2 H), 1.20–1.11 (m, 15 H), 0.68–0.61 (m, 2 H), −0.07 ppm (s, 2 H).

### Preparation of 1,1,1,3,3,6-hexachloro-5-methyl-1,3-disilahexane (24)

A 2-liter flask equipped with magnetic stirrer, condenser, pot thermometer, and addition funnel was charged with the 1,1,1,3,3-pentachloro-1,3-disilapropane/1,1,1,3,3,3-hexachloro-1,3-disilapropane mixture prepared above (1,455 g; 2.05 mol active). The addition funnel was charged with methallyl chloride (186 g; 2.05 mol). The reaction mixture was warmed to 110 °C and to initiate the reaction 20 g of the olefin was added followed by the addition of 0.5 mL of a 5 % solution of chloroplatinic acid in THF. The remainder of the olefin was added a rate such as to maintain a reaction temperature between 110 and 120 °C. After completion of the olefin addition, the reaction was heated for an additional 3 h at 130 °C. Distillation of the product directly from the reaction vessel provided 412 g (60 %) of the title dipodal silane. B.p. 89–91 °C/0.4 mmHg; density, 20 °C: 1.380 g/cc.

### Preparation of 1,1,1,3,3-pentaethoxy-6-chloro-5-methyl-1,3-disilahexane (25)

The product from above was reacted according to the representative esterification procedure to give a 60 % yield of the title organosilane. B.p. 115–117 °C/0.5 mmHg; density, 20 °C: 1.020 g/cc; ^1^H NMR (400 MHz): *δ*=3.85–3.72 (m, 10 H), 3.56–3.50 (m, 1 H), 3.39–3.33 (m, 1 H), 2.15–2.05 (m, 1 H), 1.23–1.15 (m, 15 H), 1.07 (d, 3 H), 0.9–0.83 (m, 1 H), 0.68–0.61 (m, 1 H), −0.03 ppm (s, 2 H).

### Preparation of 3-[2-(aminoethylamino-5-methyl)]-1,1,1,3,3-pentaethoxydisilahexane (26)

A 500 mL flask suitably equipped was charged with 1,1,1,3,3-pentaethoxy-6-hexachloro-5-methyl-1,3-disilahexane (96.8 g; 0.25 mol) and ethylene diamine (75.1 g; 1.25 mol). The reaction mixture was heated to 110 °C for 16 h and 25 g (0.54 mol) of ethanol added and the bottom layer separated and wiped-film distilled to yield 81 g (89 %) of the title dipodal silane. B.p. (est) 130–140 °C/0.5 mmHg; density, 20 °C: 0.990 g/cc; ^1^H NMR (400 MHz): *δ*=3.83–3.69 (m, 10 H), 2.76–2.71 (m, 2 H), 2.68–2.59 (m, 2 H), 2.51–2.45 (m, 1 H), 2.40–2.32 (m, 1 H), 2.88–2.78 (m, 1 H), 1.34 (br s, 3 H), 1.22–1.11 (m, 15 H), 0.95 (d, 3 H), 0.79–0.71 (m, 1 H), 0.56–0.48 (m, 1 H), −0.06 ppm (s, 2 H); ^29^Si{^1^H} NMR (400 MHz, CDCl_3_): *δ*=−45.62, −8.50 ppm.

### Methodology for deposition of silanes

Borosilicate glass slides (Schott North America) were acid etched before silane treatment by dipping in 4 wt. % HCl for 45 min before washing with 1) deionized water (5 mL), 2) ethanol (5 mL), and 3) acetone (5 mL). The slides were dried under N_2_ and treated with the various silanes immediately.

A 2 wt. % solution of *n*-decyltriethoxysilane (**6**) was prepared in cyclohexane and a small amount of deionized water (less than 0.5 wt. %; containing enough glacial acetic acid so that the pH 3–5) was added with stirring. The stirred silane solution was allowed to hydrolyze for 5 h prior to dipping the cleaned glass slides into the stirred solution. After 20 h, the glass slides were removed and rinsed with cyclohexane, and dried under N_2_ before being put into an oven at 110 °C for 30 min. The glass slides were cooled to room temperature in a desiccator and the water contact angles were measured. The contact angle measurements were carried out on a Ramé–Hart 100 goniometer. Multiple sessile drop contact angle (advancing and receding) measurements were carried out for each silane and the average numbers (minimum of 6 measurements) are reported. Glass slides were also prepared for silanes 1,2-bis(trimethoxysilyl)decane (**8**), 1,1,1,3,3-pentamethoxy-1,3-disilatridecane (**9**), and 1,10-bis(trimethoxysilyl)decane (**7**), and the respective water contact angles were measured following the same procedure.

### Methodology for contact angle durability studies

The durability of the silane coating was assessed by static immersion tests at room temperature in deionized water, 6 m HCl, 1 m NH_4_OH, and 3.5 wt. % aqueous NaCl. Each slide was removed from the solution, rinsed with water, and dried under nitrogen before being put into an oven at 110 °C for 30 min. On cooling to room temperature, the water contact angles were measured. After each contact angle measurement, the treated slides were re-immersed in their parent baths (deionized water, 6 m HCl, 1 m NH_4_OH or 3.5 wt. % aqueous NaCl). The process was repeated for all the samples at specified intervals for ca. 140 days or until the coatings were completely delaminated.
